# Revealing the Dark Side of Portlandite Clusters in Cement Paste by Circular Polarization Microscopy

**DOI:** 10.3390/ma9030176

**Published:** 2016-03-08

**Authors:** Oğuzhan Çopuroğlu

**Affiliations:** Section Materials and Environment, Faculty of CEG, Delft University of Technology, Stevinweg 1, Delft 2628CN, The Netherlands; o.copuroglu@tudelft.nl

**Keywords:** portlandite, circular polarization, image analysis, concrete microscopy, characterization, optical microscopy

## Abstract

Plane and crossed polarization are the two standard light modes in polarized light microscopy that are widely used to characterize crystalline and amorphous phases in cement-based materials. However, the use of the crossed polarized light mode has been found to be restrictive for studying birefringent phases quantitatively due to the extinction phenomenon that arises depending on the crystal orientation. This paper introduces circular polarization microscopy as an alternative technique to overcome the extinction problem during the examination of cementitious materials’ microstructure with optical microscopy. In order to evaluate the feasibility of this technique, selected optical and micromorphological features of portlandite clusters were investigated in cement paste. Image analysis results showed that compared to the conventional crossed polarization technique, circular polarization offers significant advantages when portlandite quantification is of interest, and it stands out as a promising low-cost alternative to backscattered electron microscopy.

## 1. Introduction

Quantitative modal analysis of cementitious materials is an essential methodology for understanding and evaluating the actual condition of the materials, as well as the history of their microstructural development [1]. Numerous microscopic, spectroscopic and macroscopic techniques have been put into good use for the characterization of cement-based materials successfully [2]. As many times demonstrated, each characterization technique is known to show strengths and weaknesses in analyzing materials. Therefore, the need for research into the feasibility of adopting alternative techniques for cement-based materials’ characterization continues to grow.

The main interest in the microstructural characterization has been directed to understand the qualitative/quantitative modal composition of phases and their chemical/mineralogical features. This information is often necessary to understand the hydration kinetics of existing and novel cement types. Furthermore, forensic materials engineering also benefits from obtaining accurate phase characteristics of cementitious materials.

An example among the phases that critically influence the performance of a cement-based material is Ca(OH)${}_{2}$, of which the crystalline variant is known as portlandite. Portlandite is quantitatively the most important crystalline hydration product, which is known to affect the carbonation and corrosion resistance of concrete [3]. In blended cement systems, the role of portlandite in carbonation shrinkage and surface durability is critical [4,5]. A direct relationship between its size, quantity and distribution and the original concrete mix characteristics (e.g., W/C) makes analyzing this phase valuable for forensic concrete microscopy [6,7].

The most popular and perhaps equally successful methods for quantification of portlandite in cement paste are the thermogravimetric methods (TG) [8,9], electron microscopy with backscattered electron detection (SEM-BSE) [10,11,12] and X-ray powder diffraction (XRD) with Rietveld analysis [13]. While TG and XRD analyses provide portlandite content as weight-percent, SEM-BSE image analysis through grey scale histogram thresholding is valuable for obtaining the approximate volumetric proportion, size and distribution of this phase within the cement paste matrix. Optical microscopy, especially polarized light microscopy, has been widely used for qualitative evaluation of the portlandite content [14,15]. Because of its moderately high birefringence (δ=0.027), portlandite stands out clearly within the optically-isotropic, C-S-H-rich matrix when examined in crossed-polarized light (XPL) mode. However, the main reason why XPL microscopy has not become a go-to technique for quantitative analysis is the full or partial extinction of an unknown portion of portlandite crystals under XPL mode [16]. The only workaround to this problem is to rotate a given portlandite cluster so that its previously extinct portion becomes visible. However, this approach is not practical when quantitative analysis of the entire portlandite clusters is of interest within a specified field of view, as some of the crystals will appear extinct no matter which angle of stage rotation is set.

**Figure 1:**
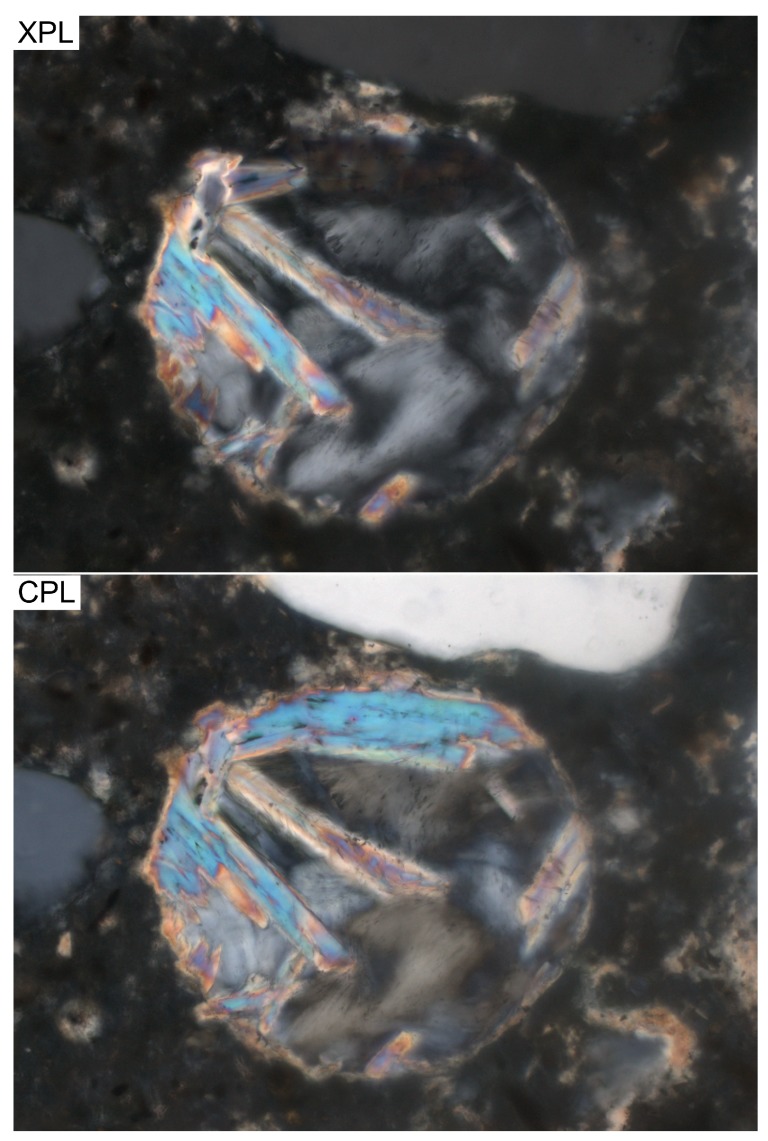
Photomicrographs of the same portlandite cluster in an air void under crossed polarization (**XPL**); and circular polarization (**CPL**) light modes. The field of view is 350 μm.

However, conventional linear crossed polarization, XPL, is not the only option to analyze birefringent phases with optical microscopy. Besides linear crossed polarization, circular polarization is another available technique through which one can examine birefringent phases, such as portlandite [17]. The most striking advantage of using circular polarization is the ability to obtain the highest-order interference colors possible of birefringent phases, regardless of the microscope rotary stage orientation. In Figure 1, an air void filled with portlandite crystals is shown under the crossed (XPL) and circular polarized light (CPL) modes. It can be seen that using the latter reveals the previously extinct (dark) portion of the cluster under the XPL mode, without changing the rotary stage angle.

**Figure 2:**
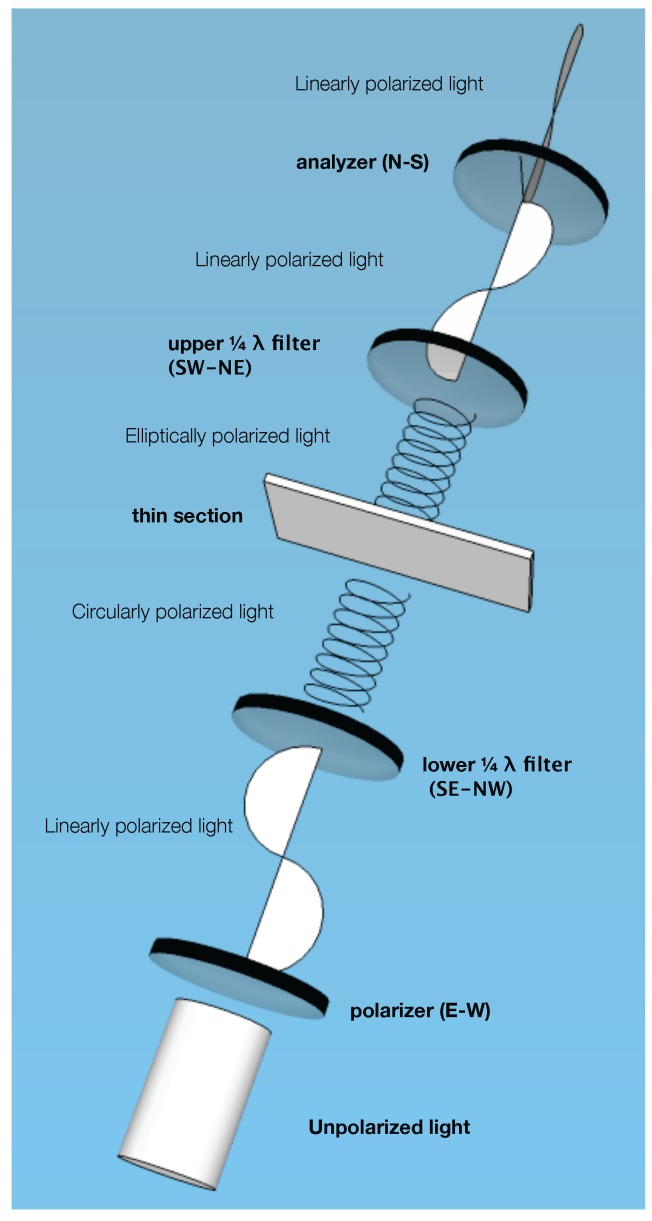
Optical filter configuration of a typical circular polarization microscope for transmitted light illumination. The operator to specimen direction is considered South-North (S-N). Other directions in the figure are E-W: East-West, SE-NW: Southeast-Northwest, SW-NE: Southwest-Northeast, N-S: North-South.

The circular polarization technique is especially suitable for the crystalline phases with a birefringence of about 0.010 and higher because the interference colors obtained by this technique match the interference color charts accurately [17,19]. This technique has, for curious reasons, attracted negligible attention from researchers, perhaps due to requiring an unorthodox setup of two additional quarter-wave (¼ *λ*) filters, although both upper and lower ¼ *λ* filters are readily available from the main microscope manufacturers. One of the quarter-wave filters is placed between the polarizer filter and the specimen, and the other quarter-wave filter is inserted in the filter slot above the objective turret. It is essential that these two additional quarter-wave filters’ slow directions are positioned at right angles. The orientation of the filters and the overall microscope setup for this technique are given in Figure 2. Although limited, notable use of this technique can be found in life sciences research [20].

In this paper, the feasibility of circular polarization microscopy in cement-based materials research is explored through a comparison study between XPL and CPL microscopy on portlandite quantification in cement paste. Additionally, the performance of CPL microscopy was briefly compared to SEM-BSE, keeping in mind that these two techniques consider different material volumes for image rendition.

## 2. Experimental Section

### 2.1. Equipment and Specimen Preparation

For the experimental study, cement paste samples were prepared with a W/C of 0.60 using a portland cement of type CEM I 52,5 N, *cf.* the European standard EN197-1 [18] (Table 1). A high W/C ratio was chosen in order to obtain relatively larger portlandite clusters. After mixing, casting and closing the ϕ30 mm×50 mm cylindrical molds, the specimens were horizontally placed on a special setup for keeping the specimens revolving at a rate of 4 rpm for about 12 h. Thereafter, the cylinders were kept in a fog room until they were 28 days old. At the end of the curing period, polished thin sections were prepared for the microscopy study. During all cutting, sectioning and polishing processes, lab-grade ethanol was used as the coolant liquid. Before proceeding with the polished thin section production, small cuts of cement paste specimens were impregnated with low-viscosity epoxy without any fluorescent pigment, in order to avoid possible interference from the epo-dye color. Standard thin sections of 30 μm (including the mounting epoxy) were produced by a semi-automatic thin sectioning machine. Measurements with a Berek compensator yielded a thickness of about 27 μm cement paste and about 3 μm mounting glue.

After the production, the thin sections were further polished to be used in the electron microscope, as well as to ensure maximum sharpness where optical plane and thin section surfaces coincide. The surface polishing protocol included grinding the thin sections briefly with a #1200 SiC grinding paper, which was followed by the polishing stage accomplished by 6, 3, 1 and 0.25 μm diamond paste. Before mounting the removable cover glass, the specimen surfaces were cleaned briefly by ethanol in an ultrasonic bath.

For the transmitted light microscopy, a Leica DM2500P optical microscope (Leica Microsystems GmbH, Wetzlar, Germany) equipped with linear and circular polarization filters and semi-apochromat (fluorite) objectives were used. The contrast and resolution of the microscope was set-up according to the Köhler illumination principles. Optical photomicrographs from the thin sections were acquired with a Leica DFC310FX digital camera (Leica Microsystems GmbH, Wetzlar, Germany) at 1392×1040 uninterpolated resolution for image analysis and publication. All image analyses were carried out on lossless tagged image files (TIF).

For the electron imaging, a Philips XL30 environmental electron microscope (Philips BV, Eindhoven, The Netherlands) in backscattered electron mode (BSE) was used under Hi-Vac chamber condition and at a 15-kV accelerating voltage. The polished specimens to be analyzed were carbon coated in a Leica EM CED030 carbon evaporator (Leica Microsystems GmbH, Wetzlar, Germany) at a thickness of about 10 nm in order to minimize the charging effects.

For the acquisition of the image fields with the optical microscope, a ×20/0.50 (magnification/numerical aperture) objective was chosen. A total of 4×5 images were sequentially acquired and analyzed. This procedure resulted in images with a total resolution of about 4768×3392, representing an area of 1208×859 μm${}^{2}$. The same number of image fields were acquired through SEM-BSE imaging, covering a total specimen area of 1819×1204 μm${}^{2}$.

In order to study the effect of thin section thickness on the portlandite quantification, a single thin section of a W/C = 0.60 cement paste was gradually ground down to 60, 51, 42, 38, 34, 31 and 27 μm. At each thickness, the same field of view was acquired by CPL imaging.

The cement paste used in this study was further characterized by thermogravimetric analysis (TGA). At the end of the 28-day curing period, pieces of the paste samples were ground by mortar and pestle and immersed in isopropanol for about 30 min. Thereafter, the samples were washed by diethyl ether and placed in a 35 ${}^{\circ }$C oven for about 10 min. The TGA measurements were carried out in a Netzsch STA 449 instrument (NETZSCH-GerÃ¤tebau GmbH, Selb, Germany) under argon atmosphere with a heating rate of 10 K/min up to 1050 ${}^{\circ }$C.

### 2.2. Image Segmentation of Portlandite Clusters

Optical and electron images were analyzed with FIJI, a noncommercial, platform independent image analysis software based on ImageJ [21]. All optical image analyses were carried out upon using the Datacolor ChromaCal system (Datacolor, Lawrenceville, NJ, USA) for color calibrating the photomicrographs, as well as the monitor in order to preserve color integrity during the study. The segmentation of portlandite crystals in optical XPL and CPL images was carried out by the Trainable Weka Segmentation tool, which is developed based on Weka data mining principles [22]. This technique uses a number of machine learning algorithms upon defining color- and texture-based classes of the phases of interest. In this study, Neighbors training feature was preferred for segmentation training among a set of various features because of the low out-of-bag error it produced.

**Figure 3:**
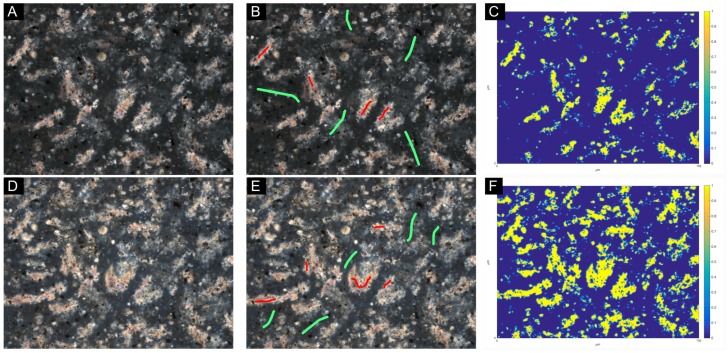
Portlandite cluster segmentation on optical photomicrographs with Trainable Weka Segmentation. Top row: (**A**) crossed-polarized light (XPL) image of the cement paste, W/C= 0.60; (**B**) an example class annotation for the Weka segmentation training; (**C**) segmented XPL image. Bottom row: (**D**) circular polarized light (CPL) image of the same field of view as the XPL; (**E**) an example class annotation for Weka segmentation training; (**F**) segmented CPL image. Note: the color scale denotes the probability of pixels segmented as portlandite, based on the classifier used. The field of view of all images is 705 μm.

When defining classes on the CPL and XPL photomicrographs, only the regions corresponding to the interference colors of portlandite crystals were considered, which corresponded to a retardation value of about 350 nm or higher. One problem with this approach was overlooking smaller portlandite clusters with a thickness of less than about 10 μm, as their interference colors were then observed as first order white or light grey, which interfered with other similar looking components, such as alite or ettringite. Similarly, some belite grains showed comparable interference colors to the portlandite clusters, but their distinct globular habit allowed an easy manual isolation. For training the segmentation tool, a ground-truth reference segmentation was prepared manually on a single 400×400 pixel cropped photomicrograph. Based on the manual segmentation, a classifier was generated using the Neighbors training feature (Out-of-bag error = 0.971%), and this same classifier was applied on all image fields to ensure consistency in the segmentation results. In Figure 3, a portlandite cluster segmentation example is shown for XPL and CPL photomicrographs of the same field of view. It should be noted that the author does not claim that this segmentation method is the most suitable option, due to the known limitations of the image analysis techniques in general. However, for a sound comparison between the performances of XPL and CPL imaging, the technique adopted in this paper was found to be reproducible.

Next to the trainable image segmentation, the point counting technique was applied on the CPL image fields. This technique is often recommended as an alternative to image analysis when the microstructural features are complex. The goal for point counting analysis was to see the comparability between the automatic segmentation and the manual point counting. A total of 6000 points were counted on an area of 1208×859μm${}^{2}$. This number was determined by using the formula:

(1) $$P_{t}=0.453\times \frac{\left(1-V_{v}\right)}{\left(V_{v}\times E^{2}\right)}$$

where $V_{v}$ is the coverage of the phase of interest as a fraction of 1; and *E* is the desired margin of error as a percentage [23]. In this study, $V_{v}$ and E were chosen as 0.15% and 2%, respectively.

In point counting applications, portlandite clusters were classified under two different classes based on their visible interference color under the CPL mode. The first class (*i.e.*, CPL-color) denotes the portlandite clusters with a retardation color higher than the first order white, and the second class (*i.e.*, CPL-white) denoted portlandite clusters showing first order white on the interference color chart.

## 3. Results

### 3.1. Thermogravimetric Characterization of the Cement Pastes

**Figure 4:**
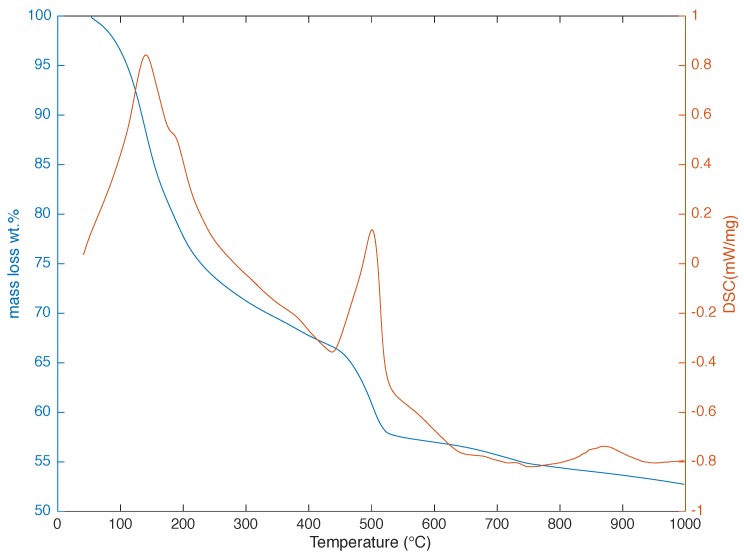
TGA (blue) and DSC (orange) curves of the cement paste used in this study.

Thermogravimetric analysis results are given in Figure 4. From the thermogravimetric data, portlandite content of the pastes with W/C = 0.60 was computed as 17.9 wt %.

### 3.2. Automatic Image Segmentation and Point Counting

In Table 2, the results of point counting and automated image segmentation of the CPL, XPL and SEM-BSE photomicrographs are given. The portlandite content results from the CPL point counting measurements were further classified as CPL-color (retardation, Δ > ∼350 nm) and CPL-white (Δ < ∼350 nm). The results showed that the image analysis and point counting methods yield comparable portlandite contents when CPL and SEM-BSE were considered. On the other hand, portlandite content deficiency on the XPL image analysis is significantly high compared to what CPL and SEM-BSE analyses deliver.

**Figure 5:**
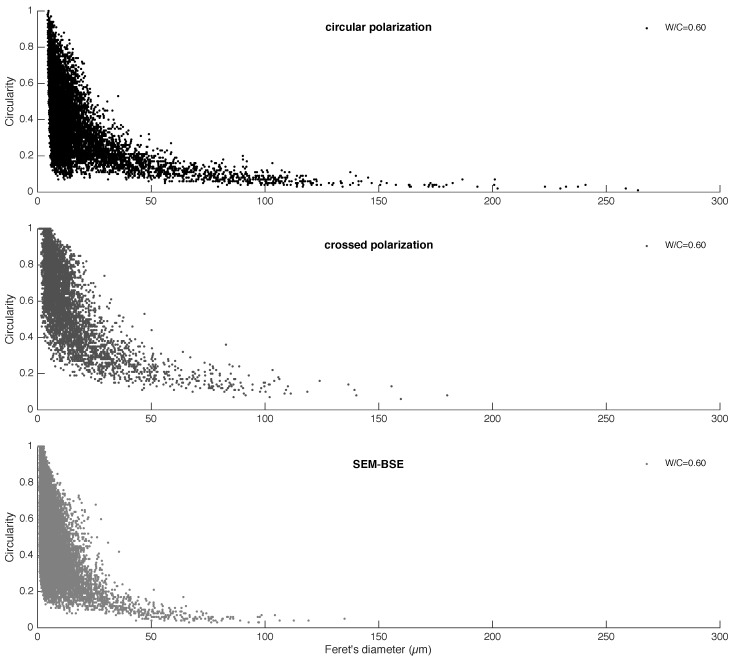
Micromorphological features of the portlandite clusters in OPCpaste with a W/C = 0.60; circularity *versus* Feret’s diameter. Based on circular polarization; crossed polarization; and SEM-BSE imaging.

The discrepancy between the different imaging techniques was further examined by investigating the morphological characteristics of the portlandite clusters through image analysis. In Figure 5, circularity *versus* Feret’s diameters of individual portlandite clusters are plotted. The results showed that the maximum Feret’s diameter of the portlandite clusters was measured as high as about 270 μm on the CPL images, while XPL images allowed the detection of portlandite clusters of about 180 μm. In SEM-BSE, the largest Feret’s diameter detected was about 140 μm. The circularity of individual clusters in this study was calculated as:

(2) $$C=4\pi (\frac{A}{p^{2}})$$

where *C* is the circularity index; *A* is the area of a portlandite cluster; and *p* is the perimeter of a portlandite cluster. The higher the circularity value of a cluster, the more its shape approaches a perfect circle. In the plots, the largest Feret’s diameter measured was used for a given cluster.

### 3.3. Effect of the Rotary Stage Orientation

**Figure 6:**
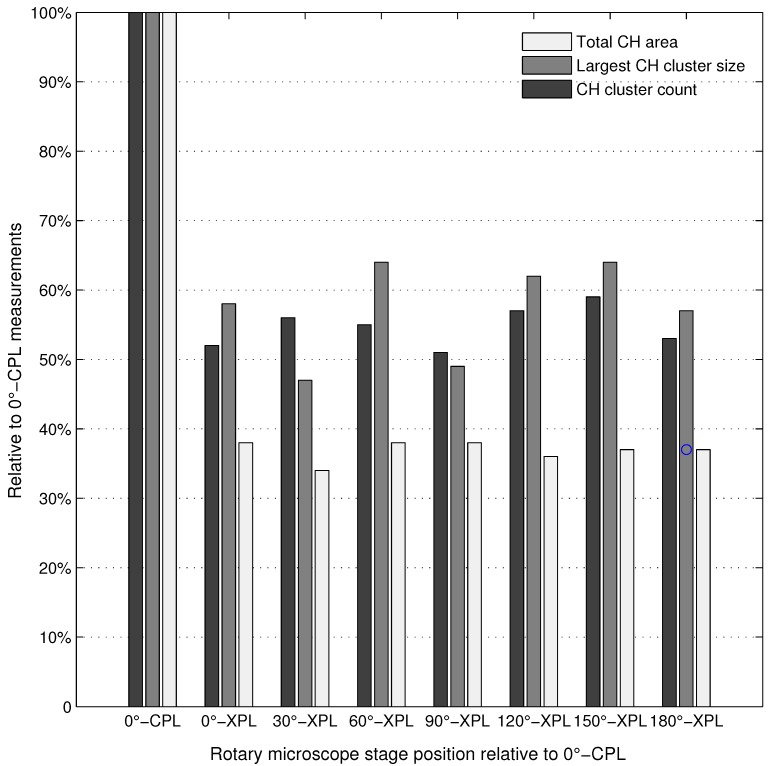
Comparison of selected portlandite metrics on the XPL images acquired at different rotary microscope stage positions relative to the measurements on the CPL image.

The comparison of the XPL and CPL imaging performances regarding portlandite cluster quantification was further evaluated by acquiring photomicrographs under XPL and CPL light modes at various rotary stage angles. The XPL and CPL image fields were automatically analyzed using the identical Weka classifier, and the total portlandite area, the largest portlandite cluster size and the total cluster count results relative to the CPL measurements are reported in Figure 6.

### 3.4. Effect of Thin Section Thickness

**Figure 7:**
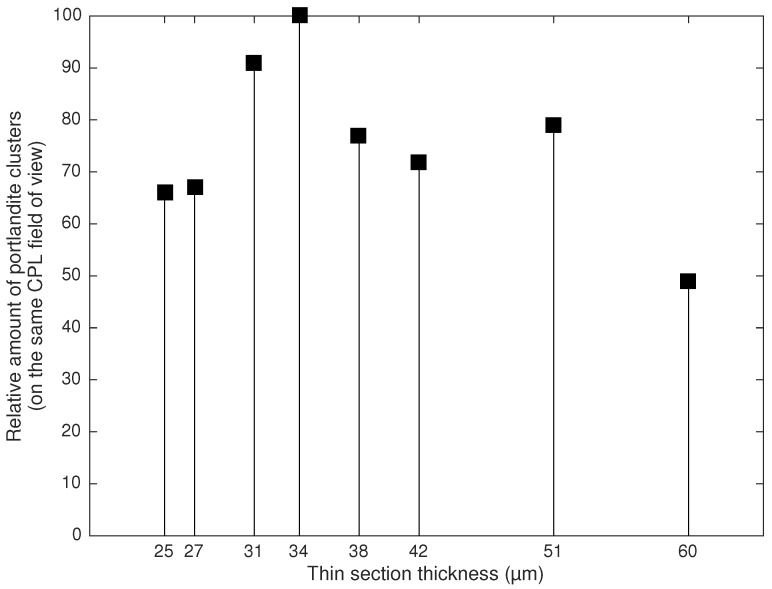
Effect of thin section thickness on the portlandite content estimation. The results are based on point counting and are relative to the 34 μm section thickness.

As portlandite cluster thickness within a thin section affects the interference colors produced, the sensitivity of portlandite color in relation to the thin section thickness was investigated. Figure 7 shows the relative point counting results of portlandite clusters on the same field of view where the section thickness had been gradually decreased from 60 μm down to 25 μm. It should be noted that concrete microscopists often prefer using 20 to 25 μm section thicknesses to assist with the study of the matrix. According to the results, the highest portlandite cluster vol% was found on the 34 μm thickness thin section. The 31 μm thin section revealed 91% of the highest observed vol%. Lower thin section thickness values rendered a significantly greater amount of first order white, which caused confusion for distinguishing portlandite from other available cement paste phases with a similar interference color. The poor transparency of the thicker sections revealed about 25% lower portlandite content up to 51 μm, while the observable content dropped drastically at about 60 μm.

## 4. Discussion

The portlandite clusters in the cement paste exhibited a hyperbolic relationship between the circularity and the Feret’s diameter values based on the XPL, CPL and SEM-BSE image analysis (see Figure 5). A similar relationship was shown earlier on the SEM-BSE images by Gallucci and Scrivener [24], while the current study shows that optical microscopy in CPL mode is also a capable technique to characterize the micromorphological features of portlandite clusters in ordinary portland cement paste.

### 4.1. Effect of Image Rendition Volume

It was interesting to see the CPL mode revealing a remarkably higher number of clusters, especially with a Feret’s diameter of higher than about 50 μm in comparison with the XPL and SEM-BSE modes. Besides the fact that CPL reveals around 50 vol% more portlandite clusters compared to the XPL mode, the main reason for detecting a higher number of larger clusters is likely due to the specimen volume represented by the CPL mode when rendering an image field. In cement paste, especially large portlandite clusters are often observed in a polycrystalline (two or more crystals) configuration, which can be observed partially when XPL mode is used and fully when CPL mode is activated (Figure 1).

**Figure 8:**
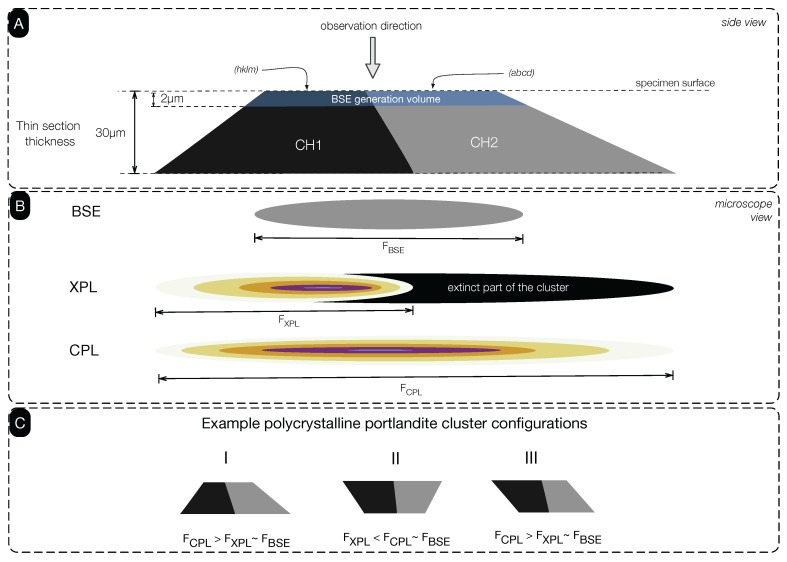
(**A**) Appearance of a hypothetical portlandite cluster with two adjacent crystals (Type I), side view in a 30-μm section; (**B**) Rendition of the cluster under SEM-BSE, XPL and CPL modes as observed by the microscopist; (**C**) Example polycrystalline portlandite cluster configurations found in portland cement pastes.

As illustrated in Figure 8A, let us consider a hypothetical portlandite cluster formed by two adjacent portlandite crystal grains (CH1 and CH2). Coexisting with the thin section surface plane, these crystal grains would have crystallographic planes that are perpendicular to the observation direction, denoted as (hklm) for the crystal CH1 and (abcd) for the crystal CH2. Because of having two different crystal orientations in the cluster, at a given rotary stage position, one of these crystals may appear extinct or near-extinct under XPL mode and become undetectable to the observer (*i.e.*, CH2 in our example). In contrast, the CPL mode would reveal the entire cluster regardless of which rotary stage position was chosen (in rare cases, one of the optical axes of a crystal grain would coincide with the observation direction, which may cause the crystal to be extinct even in CPL mode). Consequently, this would lead the CPL mode to allow the observation of a larger Feret’s diameter compared to the XPL (Figure 8B). On the other hand, because SEM-BSE acquisition renders a portlandite cluster image from a shallow depth of about 1 to 2 μm [25], again, a limited portion of the portlandite cluster would be detected. Considering the Type I cluster configuration as per the guide given in Figure 8C, Feret’s diameter of a given cluster as detected by the three acquisition methods would be observed as:

(3) $$F_{CPL}>F_{XPL}∼F_{SEM-BSE}$$

where $F_{CPL}$, $F_{XPL}$ and $F_{SEM-BSE}$ are the maximum Feret’s diameters detected in CPL, XPL and BSE image acquisition modes.

Arguably, polycrystalline portlandite clusters may form in various configurations, e.g., Type II and Type III next to Type I (Figure 8C). Observations suggest that portlandite formations typically occur as small single grains and to a lesser extent are observed in larger portlandite clusters, which show a combination of all of the aforementioned configurations. It should be noted that the exact proportions of the cluster types are unknown. If we take the circularity Feret’s diameter scatter plots in Figure 5 as a basis, it is plausible to suggest that Types I and III as a whole would occur relatively more often than any other possible polycrystalline cluster types.

However, looking exclusively at the total portlandite quantification performance by image analysis, the use of CPL mode is clearly an improvement over the conventional XPL mode and is able to deliver comparable results to the SEM-BSE technique.

### 4.2. Effect of Thin Section Thickness

Image analysis of a single CPL field of view at various section thicknesses showed that thin section thickness is critical for portlandite cluster quantification. It seems that about a 30 μm material thickness (excluding the mounting epoxy) is optimum. Thinner sections tend to increase the amount of first order white phases, which can be misinterpreted as the other phases, such as ettringite or alite. On the other hand, while thicker samples may increase the amount of detectable phases with a higher color order, the transparency of a section is compromised, which leads to poor image acquisition for quantitative analysis.

While advocating for the production of a single thin section thickness is practically not reasonable, the findings in this study emphasize the importance of producing a consistent section thickness in order to achieve reproducible analysis results.

### 4.3. CPL *versus* Other Techniques

When volumetric quantification of portlandite in cementitious materials is of interest, higher accuracy may be obtained through the use of modern techniques in electron microscopy and microanalysis. In recent years, with the advent of solid state X-ray detectors and high CPU speed, spectral imaging has become an option for image segmentation of phases on the basis of X-ray microanalysis spectra [26]. The main advantage of X-ray spectral imaging is that it does not need grey scale histogram thresholding or other image segmentation protocols, which are the main sources of error in quantitative image analysis, though spatial resolution remains as a critical parameter inherently.

However, electron microscopy and X-ray microanalysis systems, let alone the ones with the X-ray spectral imaging capability, are still expensive and not widely available outside academic institutions as of today. For many professionals, optical microscopy is arguably the only feasible option when volumetric and micromorphological characterization of cementitious materials is of interest. With the addition of circular polarization capability, polarized light microscopes can significantly be upgraded for quantitative analysis over the traditional XPL systems. Importantly, the cost of two additional quarter-wave filters is incomparably affordable compared to the alternative equipment.

As stated earlier, results from SEM-BSE and CPL imaging may not be directly comparable due to image rendition from different material volumes when the morphological characteristics of the phases are of interest. Therefore, a microscopist needs to establish ground-truth images depending on the imaging system in order to be able to calibrate and evaluate his or her image analysis quantification. Irrespective of the the image acquisition method chosen, developing a sound image segmentation protocol remains to be the greatest challenge in quantitative image analysis. Protocols, such as the Trainable Weka Segmentation, seem to be promising, provided ground-truth images are available for evaluation. Otherwise, such techniques cannot go beyond being useful for only relative comparisons based on a given classifier.

CPL microscopy stands out as a useful tool, not only for portlandite cluster analysis, but also for the quantitative characterization of other optically-anisotropic phases in cement-based materials, such as Type II and III thaumasite, carbonates, PVA fibers, as well as the main clinker phases. Additionally, its results can be used to verify computational cement hydration models, which attempt to simulate portlandite content, size and distribution at a given degree of hydration.

## 5. Concluding Remarks

Quantitative analysis of portlandite clusters in portland cement paste by circular polarization microscopy was evaluated, and its performance was compared to the conventional crossed polarized light microscopy, as well as backscattered electron imaging. Results of the experimental study showed that circular polarization microscopy offers significant improvement over the crossed polarized microscopy, and it has the potential to serve as a viable alternative to electron microscopy.

Further concluding remarks can be summarized as follows:

1. Circular polarization microscopy offers a higher accuracy for quantitative analysis of crystalline hydration products compared to the conventional crossed polarization. CPL microscopy reveals more than double the area of available portlandite clusters that XPL microscopy can resolve in a thin section.
2. Circular polarization microscopy can be effectively used to study the optical characteristics of portlandite. Due to the limitations of automatic image analysis, the point counting technique may yield higher accuracy for total portlandite content determination. However, image analysis is more favorable for grain size and dispersion analyses of portlandite clusters.
3. Image analysis results of CPL and SEM-BSE techniques may not be directly comparable when micromorphological characteristics are of interest due to image rendition from different material volumes. However, through automatic image analysis, the CPL technique appears to be capable of quantifying the total portlandite content with an acceptable relative accuracy matching that of SEM-BSE imaging.
4. The image segmentation protocol is critically important for reproducible quantitative analysis of photomicrographs. Sound ground-truth reference images are needed to evaluate the accuracy of image segmentation protocols.
5. Image segmentation based on Weka data mining principles is a promising method for accurate segmentation of portlandite clusters and possibly for the other phases in portland cement paste, as well.
6. It is fortunate that the standard thin section thickness of about 30 μm appears to be optimal for portlandite quantification in cement paste, hence allowing for the application of CPL microscopy of old thin sections, as well. Thinner sections (e.g., 20 to 25 μm) can be preferred in order to obtain better matrix clarity. In that case, proper calibration at such section thicknesses is recommended before attempting portlandite quantification.

## Figures and Tables

**Table 1:** Selected compositional data of CEM I 52,5 N(wt %).

CaO	SiO${}_{2}$	Al${}_{2}$O${}_{3}$	Fe${}_{2}$O${}_{3}$	K${}_{2}$O	Na${}_{2}$O	SO${}_{3}$	MgO	TiO${}_{2}$	Mn${}_{3}$O${}_{4}$	P${}_{2}$O${}_{5}$	Cl	Blaine (m${}^{2}$/kg)
63.98	19.76	4.93	3.16	0.54	0.27	3.17	1.92	0.28	0.11	0.77	0.04	426

**Table 2:** Image analysis and point counting results overview of the XPL, CPL and SEM-backscattered electron detection (BSE) photomicrographs (in area%). Standard deviations are given between the parentheses.

	Image Analysis			Point Counting		
**W/C**	XPL	CPL	SEM-BSE	CPL-color	CPL-white	CPL-total
**0.60**	6.6 (1.4)	18.6 (2.4)	19.7 (1.7)	13.6 (1.4)	8.8 (2.1)	22.4 (1.6)
